# Pharmacological Inhibition of polysialyltransferase ST8SiaII Modulates Tumour Cell Migration

**DOI:** 10.1371/journal.pone.0073366

**Published:** 2013-08-09

**Authors:** Yousef M. J. Al-Saraireh, Mark Sutherland, Bradley R. Springett, Friedrich Freiberger, Goreti Ribeiro Morais, Paul M. Loadman, Rachel J. Errington, Paul J. Smith, Minoru Fukuda, Rita Gerardy-Schahn, Laurence H. Patterson, Steven D. Shnyder, Robert A. Falconer

**Affiliations:** 1 Institute of Cancer Therapeutics, School of Life Sciences, University of Bradford, Bradford, United Kingdom; 2 Institute for Cellular Chemistry, Hannover Medical School, Hannover, Germany; 3 Institute of Cancer and Genetics, School of Medicine, Cardiff University, Cardiff, United Kingdom; 4 Glycobiology Unit, Cancer Center, Sanford-Burnham Medical Research Institute, La Jolla, California, United States of America; University of East Anglia, United Kingdom

## Abstract

Polysialic acid (polySia), an α-2,8-glycosidically linked polymer of sialic acid, is a developmentally regulated post-translational modification predominantly found on NCAM (neuronal cell adhesion molecule). Whilst high levels are expressed during development, peripheral adult organs do not express polySia-NCAM. However, tumours of neural crest-origin re-express polySia-NCAM: its occurrence correlates with aggressive and invasive disease and poor clinical prognosis in different cancer types, notably including small cell lung cancer (SCLC), pancreatic cancer and neuroblastoma. In neuronal development, polySia-NCAM biosynthesis is catalysed by two polysialyltransferases, ST8SiaII and ST8SiaIV, but it is ST8SiaII that is the prominent enzyme in tumours. The aim of this study was to determine the effect of ST8SiaII inhibition by a small molecule on tumour cell migration, utilising cytidine monophosphate (CMP) as a tool compound. Using immunoblotting we showed that CMP reduced ST8iaII-mediated polysialylation of NCAM. Utilizing a novel HPLC-based assay to quantify polysialylation of a fluorescent acceptor (DMB-DP3), we demonstrated that CMP is a competitive inhibitor of ST8SiaII (*K*_i_ = 10 µM). Importantly, we have shown that CMP causes a concentration-dependent reduction in tumour cell-surface polySia expression, with an absence of toxicity. When ST8SiaII-expressing tumour cells (SH-SY5Y and C6-STX) were evaluated in 2D cell migration assays, ST8SiaII inhibition led to significant reductions in migration, while CMP had no effect on cells not expressing ST8SiaII (DLD-1 and C6-WT). The study demonstrates for the first time that a polysialyltransferase inhibitor can modulate migration in ST8SiaII-expressing tumour cells. We conclude that ST8SiaII can be considered a druggable target with the potential for interfering with a critical mechanism in tumour cell dissemination in metastatic cancers.

## Introduction

Whilst crucial in the course of normal development and cell differentiation [1], abnormalities in the presentation of cell-surface carbohydrates are strongly associated with cancer progression and metastasis [2,3]. The vast range of glycosylation possibilities in the *glycocalyx* provides the tumour cell with an extensive resource for altering the nature and extent of its interactions with the local environment [4]. Simultaneously, the recognition and exploitation of enzymes responsible for the biosynthesis of tumour specific glycoconjugates involved in metastatic progression offers a large, though significantly underexplored therapeutic opportunity [5,6].

PolySia has long been recognised to be essential in steering cellular interactions during neuronal development [7,8]. PolySia is a homopolymer of *N*-acetylneuraminic acid (sialic acid, Neu5Ac), linked specifically via α-2,8-glycosidic bonds, and is negatively charged at physiological pH. The major polySia-carrier is the neural cell adhesion molecule (NCAM) [9] and addition of polySia to NCAM crucially impacts neuronal development [10]. PolySia attenuates NCAM-mediated interactions, resulting in conditions permissive for changes in cell position or shape [11]. Attachment of the polyanionic polySia to NCAM doubles the hydrodynamic radius of its extracellular part, thereby increasing the inter-membrane space. This disrupts the adhesive properties of NCAM and other cell adhesion molecules [12].

Biosynthesis of polySia is effected by two polysialyltransferases (polySTs): ST8SiaII and ST8SiaIV [13] and these two enzymes are independently able to transfer multiple Neu5Ac residues in α-2,8-linkages to acceptor *N*-glycans on NCAM. Evidence clearly demonstrates that polysialylation is a protein-specific reaction in which the polySTs initially recognise sequences on NCAM prior to effecting glycan modifications [14–17]. The polySTs show independent but overlapping expression patterns [18–20], with ST8SiaII being the prominent enzyme in embryonic development [19,21]. From heavy NCAM polysialylation during the initial stages of the perinatal and early postnatal phases, polySia levels drop dramatically soon afterwards [20,22]. By this stage, polySia is essentially absent from the body [20]. Expression in the adult, at considerably lower levels, appears confined to limited brain regions of persistent neural plasticity, where ST8SiaIV is the major enzyme [18,19,22,23]. Conversely, it is ST8SiaII that is the major driver of polysialylation in tumour cells [24], where re-expression of polySia drives metastatic behaviour in cancer [6]. ST8SiaII is thus an attractive anti-tumour target.

PolySia-expressing cancers include small cell lung cancer, neuroblastoma, pancreatic cancer, pituitary tumours, glioma and Wilms’ tumour (for a review, see Falconer et al. 2012) [6]. PolySia is strongly expressed in cancer cell lines [25–30], promotes tumour cell migration *in vitro* [31] and affects tumour cell differentiation by attenuating NCAM signalling [32]. *In vivo* studies indicate that polySia-NCAM expression is closely associated with tumour invasion and metastasis, as demonstrated with neuroblastoma [30], lung cancer [33,34], pituitary cancer [35] and glioma [36] models. The role of polySia-NCAM as a key regulator of tumour cell migration was demonstrated in neuroblastoma cells *in vitro* [37] and both siRNA knock-down of ST8SiaII and enzymatic removal of polySia by endoneuraminidase (EndoN, which specifically removes polySia from NCAM) both independently lead to abolition of cell migration in tumour cells [38]. However, it is only more recently that the molecular mechanisms underpinning the role of polySia in tumour dissemination are being understood [6,37].

The evidence for the importance of polySia in tumour dissemination of those cancers where it is expressed is now compelling. Thus far, pharmacological interrogation of this interesting target has been limited by a paucity of polyST inhibitors. Sialic acid precursor molecules (e.g. *N*-butanoylmannosamine) have been reported, but whether they inhibit polySTs through conversion to the corresponding modified CMP-Neu5Ac in the cell [39–41] or lead to *de novo* biosynthesis of modified polySia remains unclear [42,43]. We previously reported small molecule inhibitors based on CMP [44]. However, a pharmacological link between polyST inhibition, polySia biosynthesis and tumour dissemination remains to be established. In this study we use CMP as a prototype small molecule polyST inhibitor and show for the first time a correlation between inhibition of ST8SiaII and tumour cell migration.

## Materials and Methods

### Materials

All general chemicals, media and media supplements were obtained from Sigma-Aldrich (Poole, UK) unless otherwise specified. DMB-DP3 was synthesised as previously described [45]. Rabbit anti-NCAM polyclonal antibody (AB5032) which recognises all NCAM isoforms was purchased from Chemicon-Millipore (Watford, UK). Anti polySia-NCAM monoclonal antibody (mAb735) [46] was used after purification on Protein A-Sepharose (Amersham Pharmacia Biotech). EndoNA2-eGFP was kindly donated by Prof. Jukka Finne (University of Helsinki, Helsinki, Finland). EndoN was obtained from Abcys (Paris, France). Human recombinant ST8SiaII was synthesised in collaboration with Dr Edward McKenzie (University of Manchester).

### Cell lines

IMR32, SH-SY5Y and DLD-1 cells were obtained from ATCC (Manassas, USA). IMR32 and SH-SY5Y cells were maintained in Minimum Essential Medium (MEM) supplemented with Foetal Calf Serum (FCS, 10%), L-glutamine (2 mM), and sodium pyruvate (1 mM). DLD-1 was maintained in Roswell Park Memorial Institute (RPMI) 1640 medium supplemented with FCS (10%), L-glutamine (2 mM), and sodium pyruvate (1 mM). The C6-STX and C6-WT glioma cell lines [36] were grown in alpha MEM medium (VWR, Leicestershire, UK) supplemented with FCS (10%).

### Measurement of ST8SiaII inhibition

ST8SiaII activity was determined under the following conditions: MES (50 mM, pH 7.0), MgCl_2_ (5 mM), CMPNeu5Ac (500 µM), ST8SiaII (250 ng), and varying amounts of DMB-DP3 were incubated at 25^°^ C for the indicated times. The reactions were terminated by 10-fold dilution in Tris–HCl (100 mM, pH 8.0) / ethylenediamine-tetraacetic acid (EDTA, 20 mM) followed by 10 min incubation at 50^°^ C. Finally, the samples were centrifuged at 20,000 g for 10 min at 4^°^ C before analysing on a DNAPAC PA 100 analytical anion exchange column (Ex. 373 nm/Em. 448 nm). The buffer system used was: mobile phase A: H_2_O; mobile phase B: 5.0 M ammonium acetate buffer (pH 7.4). HPLC was carried out using a Waters 2695 Separations Module connected to an RF-10A spectrofluorometric detector at a flow rate of 1 mL min^-1^. To determine the *K*_i_ for CMP, the reaction was carried out as above except with different concentrations of CMPNeu5Ac in the presence of increasing concentrations of the inhibitor CMP. Reactions were analysed as previously described.

### Determination of NCAM, ST8SiaII and ST8SiaIV mRNA expression by semi-quantitative RT-PCR analysis

Total RNA was isolated from the human cells SH-SY-5Y, IMR-32, DLD-1 and rat glioma C6-STX and C6-WT cells using RNeasy incorporating a DNase I step (Qiagen, West Sussex, UK) according to the manufacturer’s instructions. Reverse transcription was performed using the RevertAidTM *H* *minus* First Strand cDNA synthesis kit (Fermentas) according to the manufacturers recommendations with the primers described below (Invitrogen). Primers were: hNCAM (sense) 5’-TGC CCA TCC TCA AAT ACA AAG C-3’; hNCAM (antisense) 5’-ATC AGG TTC ACT TTA ATA GAG TTT C-3’; rNCAM (sense) 5’-TGC TCA AGT CCC TAG ACT GGA ACG-3’; rNCAM (antisense) 5’-CTT CTC GGG CTC TGT CAG TGG TG-3’; ST8SiaII (sense) 5’-CAG AGA TCG AAG AAG AAA TCG GG-3’; ST8SiaII (antisense) 5’-GTG CTT ATT CTT CTT CAG TGG CG-3’; ST8SiaIV (sense) 5’-CTA CAT AGC CTC CTA CCT GAA G-3’; ST8SiaIV (antisense) 5’-GGA CAC TGT CAT TCA GCA TGG-3’; GAPDH (sense) 5’-GGC CAA GGT CAT CCA TGA-3’; GAPDH (antisense) 5’-TCA GTG TAG CCC AGG ATG-3’. PCR amplification: initial denaturation at 94^°^ C for 3 min followed by 22 (GAPDH), 30 (NCAM) or 35 (ST8SiaII and ST8SiaIV) cycles of 94^°^ C for 30 sec, 56^°^ C for 30 sec and 72^°^ C for 45 sec. All cycles were terminated with a 5 min incubation at 72^°^ C to ensure complete elongation prior to incubation and storage at 4° C. RT-PCR products were separated through a agarose gel (1% w/v) and scanned on a Molecular Imager Fx (BioRad, Dorset, UK) using Quantity One (v 4.6.5) software.

### Western blotting for PolySia-NCAM

Cells were lysed on ice with extraction buffer, containing Tris-HCl (50 mM, pH 8.0), NaCl (150 mM), EDTA (2.5 mM), Triton-X-100 (1%), and 1 x protease inhibitor cocktail (Sigma) for 60 min. The resulting lysate was centrifuged at 2000 g for 15 min at 4^°^ C, and the concentration of the protein concentration in the supernatant determined by Bradford assay. Samples were resolved on 6% (50 µg, polySia) and 12% (25 µg, β-actin) polyacrylamide gels and blotted onto nitrocellulose membranes (Amersham). Non-specific antibody binding was blocked via incubation in ECL blocking reagent (5%, Amersham) and the blot was probed with either anti- polySia antibody (1:3000 dilution) or mouse anti-β-actin (1:10 000 dilution; Sigma) overnight at 4^°^ C. Antibody reactivity was detected by horseradish peroxidase (HRP)-conjugated antibody and chemiluminescence using ECL-Plus (Amersham).

### Quantification of cell surface polySia-NCAM by flow cytometry

Once cells had reached approximately 70% confluency, they were treated with CMP for 24 hours and then detached to produce single cell suspension. Cells were then centrifuged for 5 minutes at 400 g and pellets were re-suspended in HBSS/medium (5 mL, 2% FCS). After spinning for 5 minutes at 400 g, cells were fixed with of 70% ice cold ethanol (1 mL) in PBS at -20^°^ C for 30 minutes followed by addition of PBS (0.5 mL). Cells were then centrifuged at 400 g for 5 minutes and the ethanol/PBS layers were removed. Cell pellets were re-suspended in EndoNA2-eGFP (75 µL, 10 µg/mL) for 60 minutes in darkness. After centrifugation for 5 minutes at 400 g, cells were washed twice with PBS to remove excess EndoNA2-eGFP followed by centrifugation for 5 minutes at 400 g. Cell pellets were re-suspended in PBS (500 µL) and samples were analysed using a BD FACSCalibur flow cytometer, using Cellquest™ software (Becton Dickinson, Oxford, UK). At least 10,000 events were analysed per sample. All steps were performed at room temperature unless otherwise stated.

### PolySia-NCAM recovery assay

Cells were seeded at 1 x 10^4^ cells/mL onto autoclaved cover slips in a six-well plate in medium (2 mL) and incubated overnight at 37^°^ C in a 5% CO_2_ humidified atmosphere. Once cells had adhered to cover slips, they were treated with EndoN (0.5 µg/ml) for (24 hours), followed by two washes in HBSS. As controls, cultures were fixed directly following EndoN treatment to confirm polySia removal. Cultures were then incubated with medium containing CMP at different concentrations for 6 hours. Following these treatments, the cultures were rinsed twice with HBSS and fixed with ice cold methanol at -20° C for 30 minutes. After air drying, they were stored at -20° C or immediately processed for immunocytochemistry. Also, to confirm polySia recovery, cultures were incubated with inhibitor-free medium for the same time periods as for the CMP treatments. The resulting slides were analysed by counting the number of polySia-expressing cells. Results were expressed as migration relative to the untreated control and as the mean ± SD of three independent experiments. A Leica DMRB microscope was utilised, with digital images captured by a Leica MPS52 camera and processed using the AcQuis image capture system.

### In vitro migration assays

Scratch assay [47]: Cells were seeded into six-well plates at different concentrations, and plates were then incubated overnight at 37^°^ C in a 5% CO_2_ humidified atmosphere. Once a confluent monolayer had formed, a straight wound of approximately 1 cm in length was created in the centre of the well using a sterile P200 pipette tip. After scratching, the media was slowly aspirated and discarded, and wells were washed with growth medium (1 mL) to remove debris and smooth the edge of the scratch. After washing with HBSS, fresh medium was added to the cells with or without compound. The plates were then incubated at 37^°^ C in 5% CO_2_ for up to 24 hours. During incubation, plates were periodically monitored using phase-contrast microscopy to determine the time required for the migrating cells to fully close the wound. Measurement of the wound was achieved by measuring the gap width at five points in the centre of the wound using an eyepiece graticule with a micrometer scale. After 24 hours, cells were washed twice with Hanks Balanced Salt Solution (HBSS) and fixed with ice cold methanol at -20° C for 30 minutes. After fixation, cells were then hydrated with two washes in PBS and counter-stained with Harris’s Haematoxylin solution. They were then washed briefly under running tap water, before the excess water was drained for 1 minute and the plates left to dry at room temperature. The resulting plates were viewed under a Nikon dissecting microscope (Nikon, C-D55230, Japan) and digital images were acquired for each sample. The extent of cell migration, expressed as percentage migration, was calculated as follows:

Migration (%) = ([Average of S_0_ - Average of S_1_] / Average S_1_) × 100%, Where S_0_: is the width of initial perimeter; S_1_: is the width of wound at time of sampling. Data were expressed as means ± S.D of three independent experiments.

Cell exclusion-zone assay [48]. Cell seeding stoppers from the Oris Cell Migration Assay Kit (Platypus Technologies, Madison, USA) were inserted into each well of a 96-well plate where they formed a tight seal to the base of the plate. Cells were then seeded at a range of concentrations by adding cell suspension (100 µL). The microplate was then incubated at 37^°^ C in a humidified atmosphere of 5% CO_2_ overnight to allow for cell adherence. Once a confluent monolayer had formed around the inserts, the stoppers were gently removed followed by careful washing with HBSS to remove any detached cells. For pre-migration controls (t_0_), the stoppers were left in the wells until the day of fixation. After washing, fresh medium (100 µL) was added to the wells followed by incubation at 37^°^ C in a 5% CO_2_ humidified atmosphere for up to 72 hours to allow cell migration. Where required, CMP at varying concentrations was added to the medium. During incubation, plates were periodically monitored using phase-contrast microscopy to examine the progression of cell migration. After re-population of the detection zone, stoppers were removed from the controls (pre-migration t_0_) and all cultures were rinsed twice with HBSS and fixed with ice cold methanol at -20° C for 30 minutes. After fixation, cells were then hydrated by two washes in PBS and counterstained with Harris’ Haematoxylin solution as above. The resulting plates were viewed under a Nikon dissecting microscope (Nikon, C-D55230, Japan) and digital images were acquired for each sample. The images were analysed by counting the empty spaces in the detection zone using a 432 point grid. Percentage migration was calculated as follows: Migration (%) = ([Average of S_0_ - Average of S_1_] / Average of S_0_) × 100%, where S_0_: numbers of empty grid spaces in the detection zone at pre-migration t_0_; S_1_: number of empty grid spaces in the detection zone at t_1_ (post-migration). Data were expressed as mean ± S.D of three independent experiments.

## Results

### CMP is a competitive inhibitor of ST8SiaII

Using NCAM, in the presence of recombinant human ST8SiaII and CMP-Neu5Ac (the substrate for polysialylation reactions), CMP (1 mM) was shown to abolish polysialylation (Figure S1). We then quantified this CMP inhibition employing a 1,2-diamino-4,5-methylenedioxy-benzene (DMB)-labelled trimer of α-2,8-linked sialic acid (DMB–DP3; where DP refers to degree of polymerisation) as an alternative acceptor for polysialylation in place of NCAM [45,49]. As a novel HPLC-based assay, this is a significant improvement on previously published methods, which indirectly measure inhibition via quantification of nucleoside release from CMP-Neu5Ac following the sialylation reaction [50,51].

**Figure 1:**
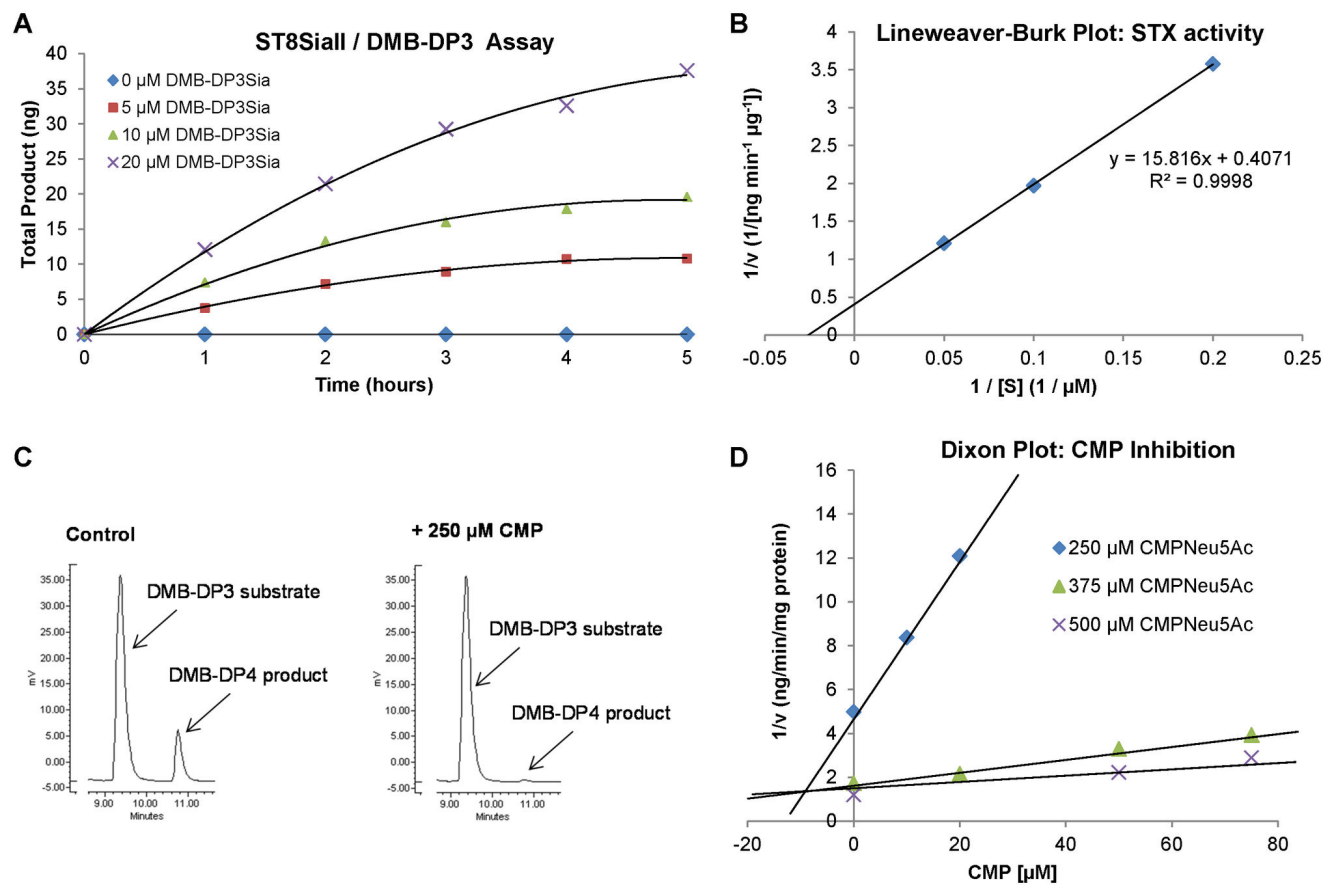
CMP is an inhibitor of ST8SiaII *in vitro*. A: Activity of human ST8SiaII with increasing concentrations of DMB-DP3 acceptor over 5 hours, as measured by HPLC. B: Lineweaver-Burk plot* generated from A.K_m_ of DMB-DP3 for ST8SiaII (derived from *x* axis intercept) was calculated as 38.9 µM. Data points are from a single determination representative of two independent experiments. C: HPLC trace extract showing that CMP (250 µM) inhibits polysialylation of DMB-DP3 (10 µM) to DMB-DP4 (ST8SiaII 250 ng; CMP-Neu5Ac 500 µM; 4 h; 25^°^ C). D: Dixon Plot* indicating that CMP is a competitive inhibitor of ST8SiaII, *K*_i_ = 10 µM (*Data points are from a single determination representative of two independent experiments).

Human ST8SiaII polysialylated DMB-DP3 (*K*_m_ = 38.9 µM, Figure 1) to DMB-DP4 as the major product, with polysialylation up to DMB-DP7 also observed. At shorter reaction times (4 h) DMB-DP4 was the only product, providing the opportunity for a single peak from which to quantify product concentrations in the presence of potential inhibitors. CMP was shown to inhibit production of DMB-DP4 in a reproducible manner (Figure 1). The activity of ST8SiaII (as measured by DMB-DP4 product formation) was assessed in the presence of varying concentrations of substrate (CMP-Neu5Ac) at each of three CMP concentrations, while the quantity of DMB-DP3 acceptor was maintained at a constant level. CMP was confirmed as a competitive inhibitor of ST8SiaII (*K*_i_ = 10 µM, Figure 1).

### CMP inhibits ST8SiaII-mediated tumour cell polysialylation

**Figure 2:**
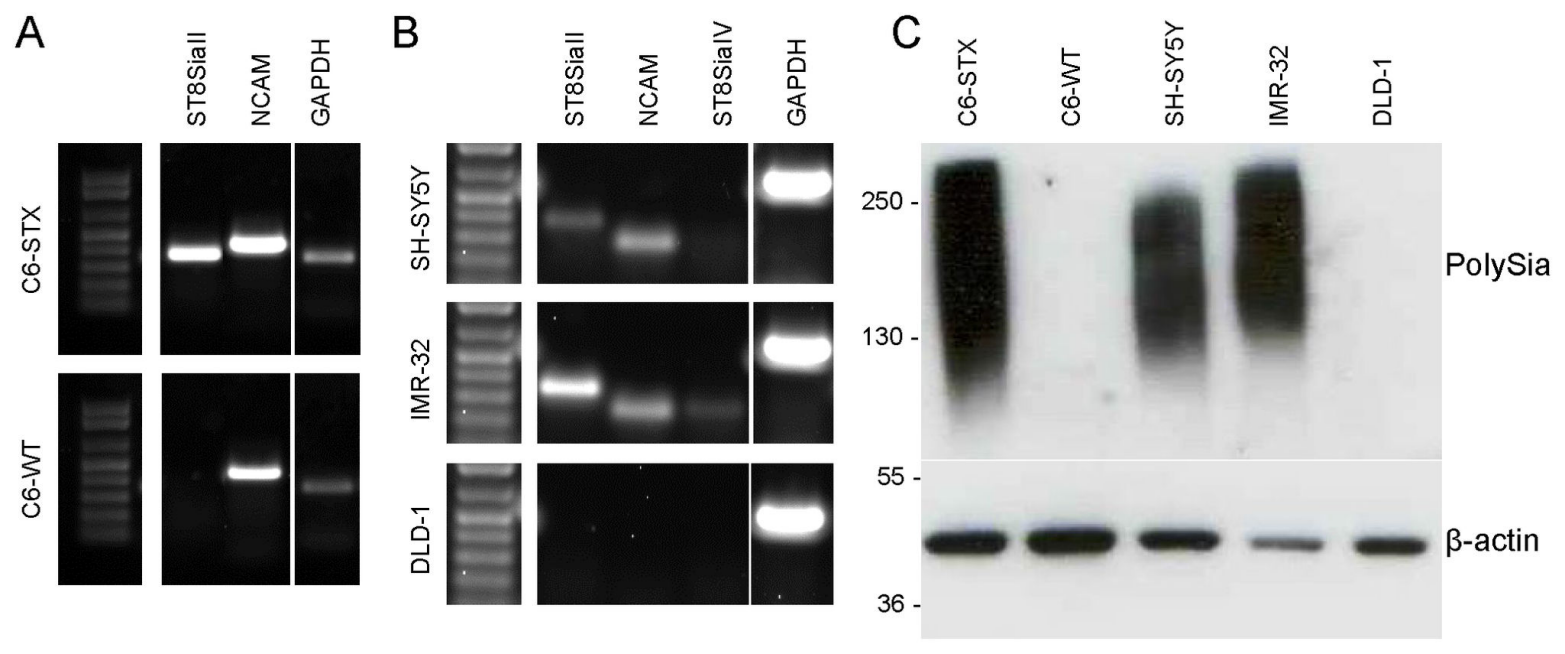
Semi-quantitative PCR showing expression of polySTs and NCAM and Western blotting showing expression of polySia-NCAM in cell lines. A: C6-STX cells express ST8SiaII and NCAM, whereas C6-WT cells express NCAM only. B: SH-SY5Y and IMR-32 cells both express ST8SiaII and NCAM. Expression of ST8SiaIV was not detected in SH-SY5Y cells, and was barely detectable in IMR-32 cells. DLD-1 cells do not express NCAM or ST8SiaII. C: C6-STX, SH-SY5Y and IMR-32 cells all express polySia, whereas C6-WT and DLD-1 cells do not.

We subsequently investigated whether ST8SiaII inhibition by CMP results in modulation of cell-surface polySia of tumour cells. We utilised isogenic C6 rat glioma cells, engineered to over-express ST8SiaII (C6-STX cells) and associated polySia [36]. Wild type (C6-WT) cells express NCAM, but not ST8SiaII and are thus polySia-negative. The mRNA expression of ST8SiaII and NCAM in transfected cell lines was confirmed by semi-quantitative PCR, while polySia and NCAM expression was established by western blot (Figure 2). Antibodies to ST8SiaII are not available, but previous studies have indicated that presence of polySia directly correlates with polyST expression [28]. We additionally evaluated polySia-expressing neuroblastoma cell lines SH-SY5Y and IMR-32. Both these human cell lines predominantly express ST8SiaII and in both cell lines NCAM is quantitatively polysialylated. We also screened for the presence of ST8SiaIV mRNA, but in accordance with literature data, SH-SY5Y cells did not express ST8SiaIV and mRNA was barely detectable in IMR-32 cells [28]. DLD-1 (colorectal adenocarcinoma) cells were selected as a negative control, as these cells did not express ST8SiaII, NCAM or polySia (Figure 2).

**Figure 3:**
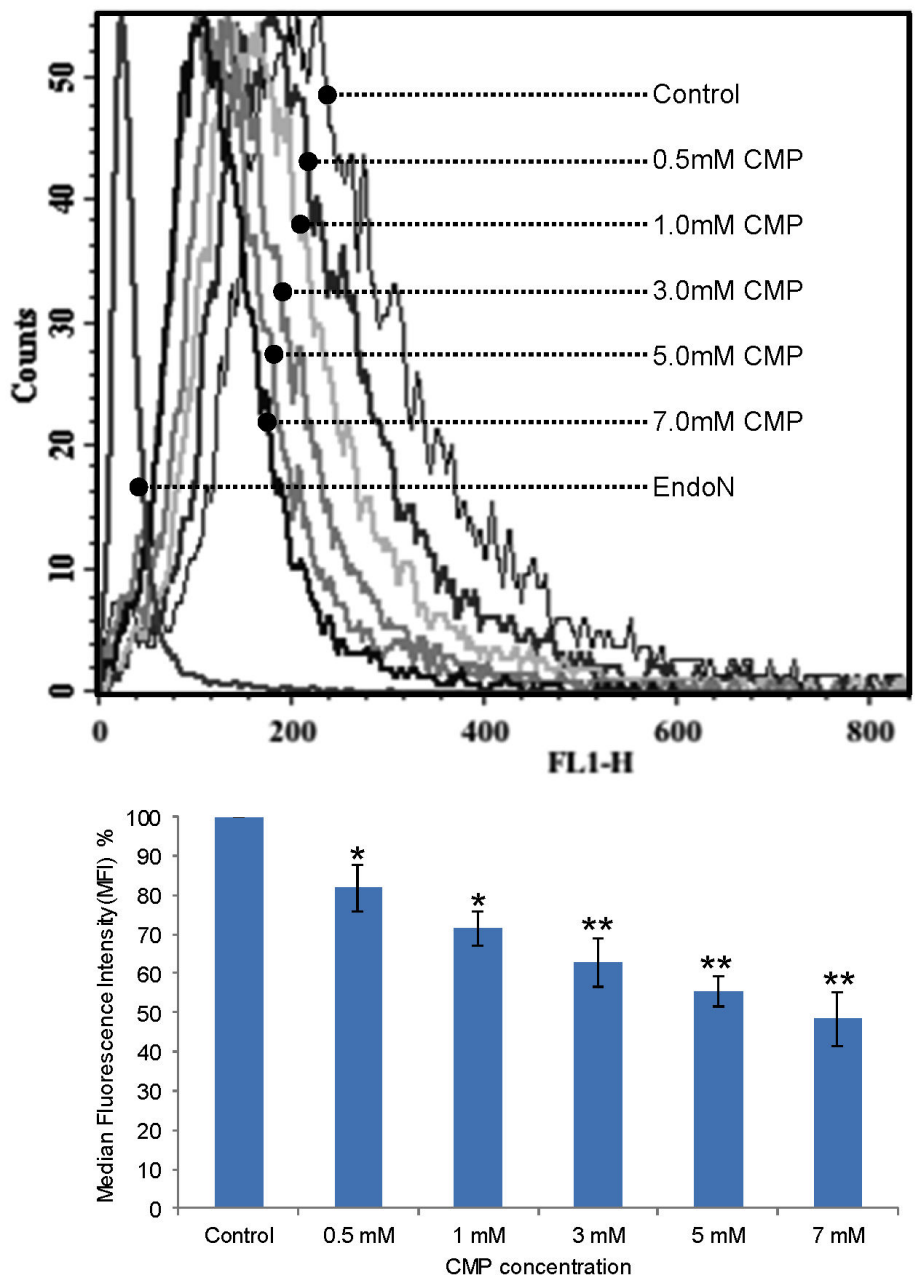
Effect of ST8SiaII inhibition on cell surface polySia expression. Upper: Flow cytometry analysis of effect of 24h exposure to increasing CMP concentrations on polySia expression in IMR-32 cells (NCAM +; ST8SiaII +; polySia +). Lower: Relative mean fluorescence intensities as a result of CMP treatment. Cells were labelled with EndoNA2-eGFP and analysed by flow cytometry. Fluorescence is expressed as a mean percentage of that observed with the control (untreated cells) ± SD of three independent experiments (* P < 0.01, ** P < 0.001). CMP causes a significant and concentration-dependent reduction in polySia expression in IMR-32 cells.

Two methods were employed to assess the effects of ST8SiaII inhibition on tumour cell polySia expression. The first method involved co-incubation of cells with CMP and measuring its effect on polySia surface-expression using flow cytometric analysis of endoNA2-eGFP, a highly specific polySia antibody-mimetic [52]. Using this method we showed that CMP inhibits NCAM polysialylation in a concentration-dependent manner in IMR-32 cells (Figure 3).

**Figure 4:**
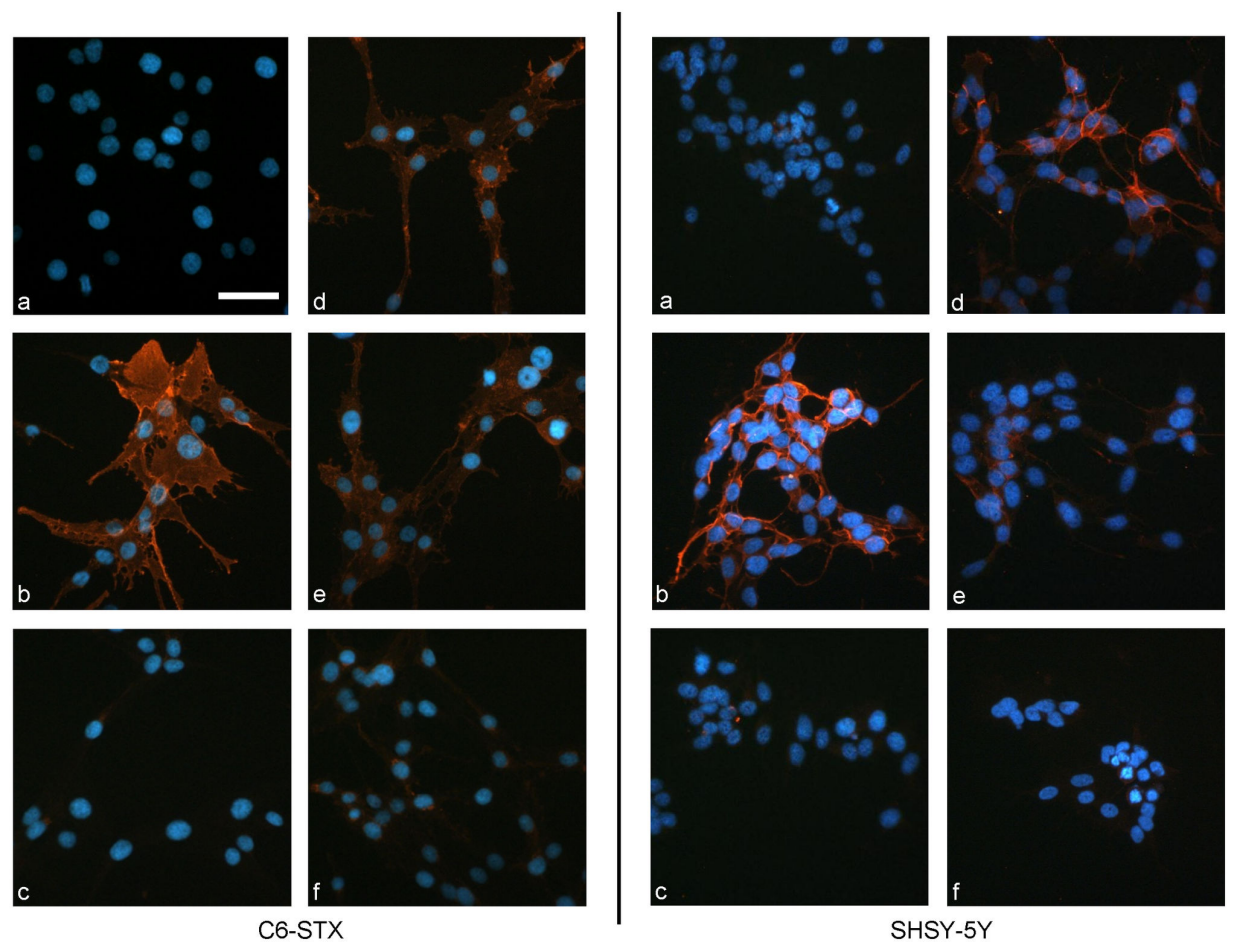
Effect of ST8SiaII inhibition on recovery of polySia expression following removal by endoN. C6-STX cells (left) and SH-SY5Y cells (right) immunolabelled with anti-polySia antibody (mAb 735) followed by incubation with TRITC-conjugated secondary antibody [PolySia orange; nuclei stained blue with DAPI]. (a) Negative control (absence of mAb 735); (b) Positive control (absence of endoN/CMP treatment) (c) Removal of polySia with EndoN (0.3 µg/mL); (d) PolySia recovery following 6 h incubation in absence of CMP; (e) PolySia recovery following 6 h incubation with CMP at 0.5 mM; (f) PolySia recovery following 6 h incubation with CMP at 5 mM. CMP clearly prevents the recovery of polySia on the cell surface following biological removal at 5 mM. Scale bar = 50 µm.

The second method employed ablation of polySia expression using endoN [53] and monitoring re-expression (recovery) in the presence of CMP. In untreated C6-STX and SH-SY5Y cells polySia expression was restored within 6 h, whereas cells treated with CMP showed a persistent absence of polySia (Figure 4). Similar results were observed with IMR-32 cells (Figure S2). No cytotoxicity was observed for any of the cell lines employed. Indeed, it was not possible to obtain an IC_50_ value from an MTT assay following 96 h exposure: CMP showed no cytotoxicity at 10 mM, the highest concentration examined.

### CMP inhibits tumour cell migration in ST8SiaII-expressing cells

**Figure 5:**
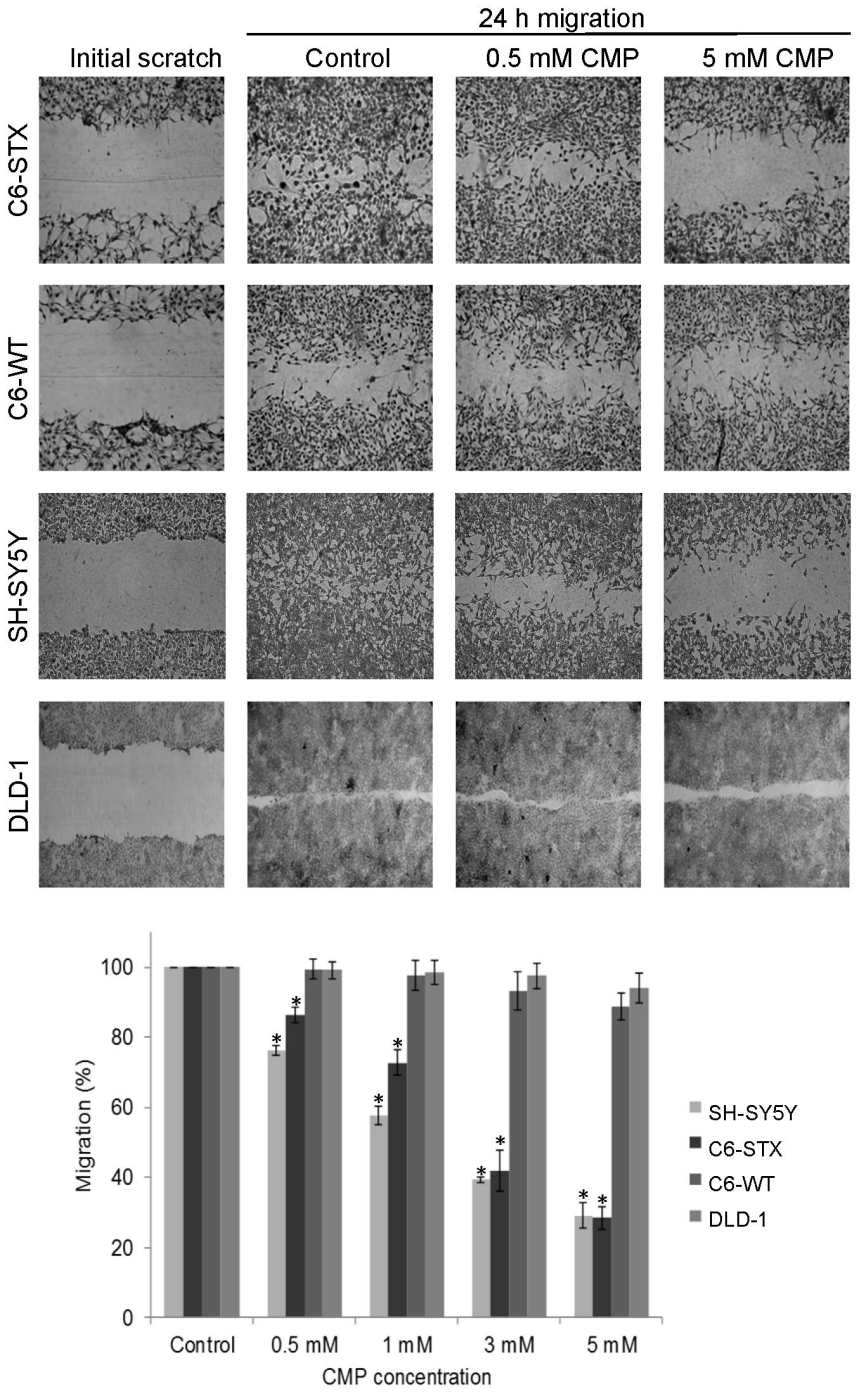
Effect of ST8SiaII inhibition on tumour cell migration. Effect of CMP treatment on the migration of C6-STX, C6-WT, SH-SY5Y, and DLD-1 cells as assessed by 2D migration assays. Confluent cell monolayers were incubated with fresh complete medium and re-population of scratched wounds after 24 h was assessed. Migration is expressed as a percentage of that observed with untreated cells for a given cell line (complete re-population). CMP treatment at all concentrations decreased the migration capacity of polySia-expressing SH-SY5Y and C6-STX cells in a concentration dependent manner. However, CMP treatment had no significant effect on the migration of C6-WT and DLD-1 cells even at high concentrations. C6-WT and DLD-1 cells do not express polySTs or polySia. Values shown are means ± S.D based on three independent experiments (* P < 0.01).

Following the demonstration that ST8SiaII inhibition by CMP leads to a concentration-dependent reduction of tumour cell-surface polySia, we investigated whether the CMP-induced inhibition of polySia expression impacted tumour cell migration. To generally document that polySia expression drives cell migration we used the rat glioma model C6, in which C6-STX cells stably express ST8SiaII [36]. Their migratory capacity was evaluated in 2D cell migration assays, specifically: (i) a ‘scratch’ assay and (ii) a cell exclusion-zone assay. C6-STX cells migrate significantly faster than C6-WT cells in 2D cell migration assays, as evidenced by the relative rates of wound closure over a 24 h timeframe (Figure 5). These effects were confirmed by biologically removing polySia from the cell surface of C6-STX cells using endoN, which resulted in a significant reduction in cell migration (Figure S3), while no effect was visible on polySia-negative C6-WT and DLD-1 cells.

Using 2D cell migration assays we demonstrated that CMP causes a significant reduction in the migration of ST8SiaII-expressing C6-STX and SH-SY5Y cells (24 h exposure, Figure 5). CMP had no effect on the migration of C6-WT (negative for ST8SiaII and polySia) or DLD-1 cells (negative for ST8SiaII, NCAM and polySia). No cytotoxicity was observed in any of the cell lines at the concentrations of CMP found to inhibit tumour cell migration.

## Discussion

The results of this study have shown for the first time that a small molecule (CMP) can modulate tumour cell migration through inhibition of polysialyltransferase ST8SiaII. The enzyme ST8SiaII was the focus for these studies, reflecting the understanding that of the two polySTs, it is ST8SiaII that acts as the major driver of polysialylation, and the associated conversion from an adhesive to a metastatic phenotype in tumour cells [24]. ST8SiaII gene expression has been shown to dramatically increase during tumour cell progression, whereas ST8SiaIV levels remain very low [54]. ST8SiaII has also been observed to regulate polySia expression in SCLC cells [55], and gene expression of ST8SiaII in neuroblastoma samples strongly correlates with stage and grade of disease and is highly prognostic of poor patient outcome [56,57]. . Additionally, oncogenic transcription factor Pax3 is closely involved in the regulation of ST8SiaII in tumour cells: engineered over-expression of this gene is known to increase ST8SiaII mRNA levels by up to four-fold, whereas ST8SiaIV mRNA is not affected [58].

We have demonstrated that CMP is a competitive ST8SiaII inhibitor, albeit with low potency, and that it modulates polySia-mediated tumour cell migration. The high (mM) concentrations of CMP required to inhibit ST8SiaII and tumour cell migration are likely to be due to the hydrophilic/lipophilic balance of the molecule limiting membrane permeability. However, our cell-based studies indicate that CMP is sufficiently lipophilic to reach the Golgi-localised ST8SiaII [59]. It is possible that ST8SiaII inhibition by CMP is rate-limited by its transport into the Golgi apparatus [44].

Glioma cells are highly invasive and migratory *in vivo* by a process shown to be dependent on polySia [36] and our studies are consistent with a role for polySia in migration of NCAM-polySia expressing tumour cells. We previously demonstrated that following injection into the brain, C6-STX cells (but not C6-WT cells) invaded the corpus callosum [36]. Our present results demonstrate that C6-STX cells migrate faster than C6-WT cells *in vitro* and thus represent a useful model to assess the anti-migratory effects of polyST inhibitors. ST8SiaII knockdown by siRNA [38] or biological removal of polySia by endoN [37,60] both indicate that ST8SiaII-mediated polySia biosynthesis is crucial for migration in polySia-expressing tumour cells, further demonstrating the significance of this target.

The precise molecular events by which polySia and NCAM modulate cell adhesion, migration and invasion have yet to be elucidated. However, key to the potential modulating effects of polySia is the impact on the homophilic and heterophilic interactions of NCAM and the complex network of intracellular signalling activated upon binding [61]. For example, polySia is known to potentiate signal transduction via FGFR and focal adhesion kinase (FAK) signalling, which in turn affects Fyn, ERK and other downstream events [62–65]. PolySia-NCAM signalling through FAK and involving both Ras-MAPK and FGFR-PLCγ-PKC has also been demonstrated [66] and a role has additionally been elucidated for the PI3K/Akt pathway in NCAM-mediated cell survival [67]. NCAM is additionally known to interact with several extracellular matrix components, including heparin, collagen, cadherins, a number of chondroitin sulfate and heparan sulfate proteoglycans [68–70]. PolySia and NCAM thus regulate cell-cell and cell-substrate interactions. Important in this context is that both factors have global effects on cellular recognition processes and signalling events that elicit changes in tumour cell behaviour beyond the direct involvement of, for example, NCAM-mediated adhesion [24,66].

Here we have shown that CMP is essentially non-toxic to cells and yet has anti-migratory activity. The question remains as to whether it is possible for a small molecule ST8SiaII inhibitor to significantly reduce tumour cell invasion and metastasis, through modulation of polySia expression.

In conclusion, these results demonstrate that CMP is a competitive small molecule ST8SiaII inhibitor and that the resultant ablation of tumour cell-surface polySia expression correlates with a significant diminution in tumour cell migration. Furthermore, we have identified a link between pharmacological suppression of ST8SiaII activity and tumour cell migration and have thus shown that ST8SiaII is a druggable target.

The challenge ahead is to develop a potent, selective small molecule ST8SiaII inhibitor and to ultimately demonstrate efficacy *in vivo*. Given that metastatic spread of tumour cells is such a progressive and multifactorial process [71], and that it remains the major cause of morbidity and mortality in cancer [72], it is clear that new approaches to therapy are vital: there are currently no anti-metastatic agents available for treatment. We firmly believe that this study has finally unlocked the potential for inhibition of ST8SiaII as an exciting and desperately needed therapeutic opportunity for metastatic cancer. We are currently investigating small molecules based on CMP to identify more potent inhibitors of ST8SiaII with benefit in preventing dissemination of polySia-expressing tumours.

## Supporting Information

Figure S1 **ST8SiaII inhibition by CMP inhibits polysialylation in an *in vitro* assay.** Expression of polySia following incubation of NCAM (50 ng), ST8SiaII (250 ng), CMP-Neu5Ac (200 µM) and increasing concentrations of CMP (as indicated). Reactions were pre-incubated for 5 mins, after which CMP-Neu5Ac was added to initiate the reactions. Incubation time: 30 min at 37^°^ C, in presence of MgCl_2_ (5 mM) in MES buffer (50 mM; pH 7); total vol. 20 µL. Polysialylation is completely inhibited in presence of 1 mM CMP.
(TIFF)

Figure S2 **Effect of ST8SiaII inhibition on recovery of polySia expression following removal by EndoN in IMR-32 cells.** IMR-32 cells immunolabelled with anti-polySia antibody (mAb 735) followed by incubation with TRITC-conjugated secondary antibody. (a) Negative control (absence of mAb 735); (b) Positive control (absence of endoN/CMP treatment) (c) Removal of polySia with EndoN (0.3 µg/mL); (d) PolySia recovery following 24 h incubation in absence of CMP; (e) PolySia recovery following 24 h incubation with CMP at 0.5 mM; (f) PolySia recovery following 24 h incubation with CMP at 5 mM. CMP clearly prevents the recovery of polySia on the cell surface following biological removal at 5 mM.
(TIFF)

Figure S3 **Effect of biological removal of polySia on tumor cell migration.** Migration of C6-STX, C6-WT and DLD-1 cells was assessed. Confluent cell monolayers were incubated with fresh complete medium and repopulation of exclusion zones was assessed after 60 h for C6-STX cells, 72 h for C6-WT cells and 120 h for DLD-1 cells. EndoNF treatment of C6-STX cells led to a highly significant reduction in cell migration (17% of control, P<0.01), but no effect on C6-WT or DLD-1 cells.
(TIFF)
