# Supplementary figures and images for: Pharmacological Inhibition of polysialyltransferase ST8SiaII Modulates Tumour Cell Migration
Source: PLoS One. 2013 Aug 9;8(8):e73366. doi: 10.1371/journal.pone.0073366 (PMC3739731; doi:10.1371/journal.pone.0073366)

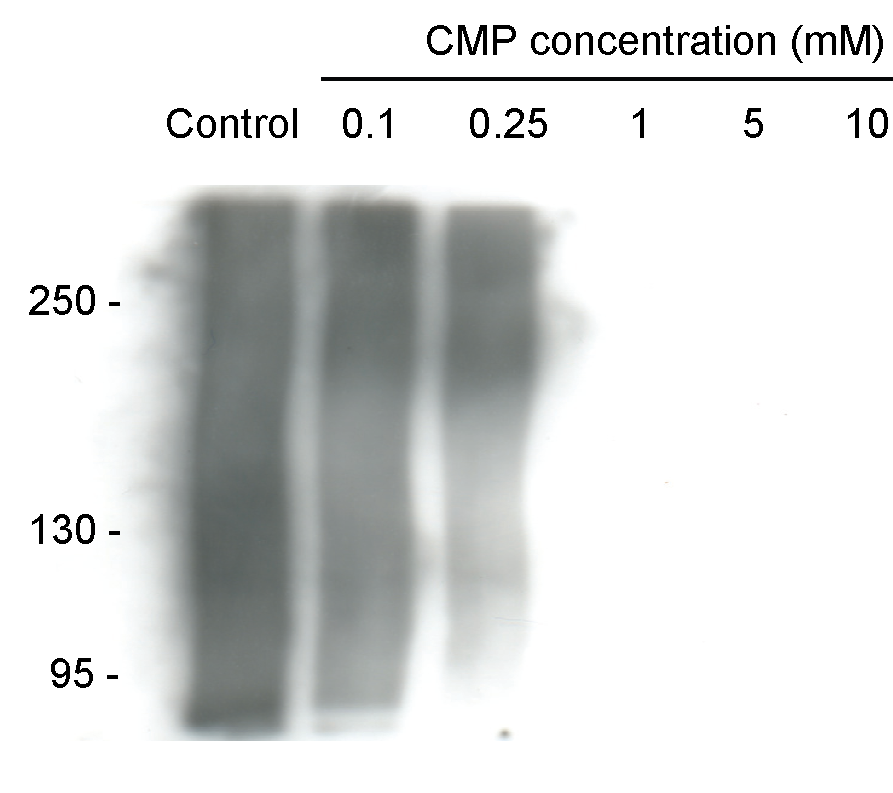

Supplement: Figure S1 — ST8SiaII inhibition by CMP inhibits polysialylation in an in vitro assay. Expression of polySia following incubation of NCAM (50 ng), ST8SiaII (250 ng), CMP-Neu5Ac (200 µM) and increasing concentrations of CMP (as indicated). Reactions were pre-incubated for 5 mins, after which CMP-Neu5Ac was added to initiate the reactions. Incubation time: 30 min at 37° C, in presence of MgCl2 (5 mM) in MES buffer (50 mM; pH 7); total vol. 20 µL. Polysialylation is completely inhibited in presence of 1 mM CMP. (TIFF) [file pone.0073366.s001.tiff]

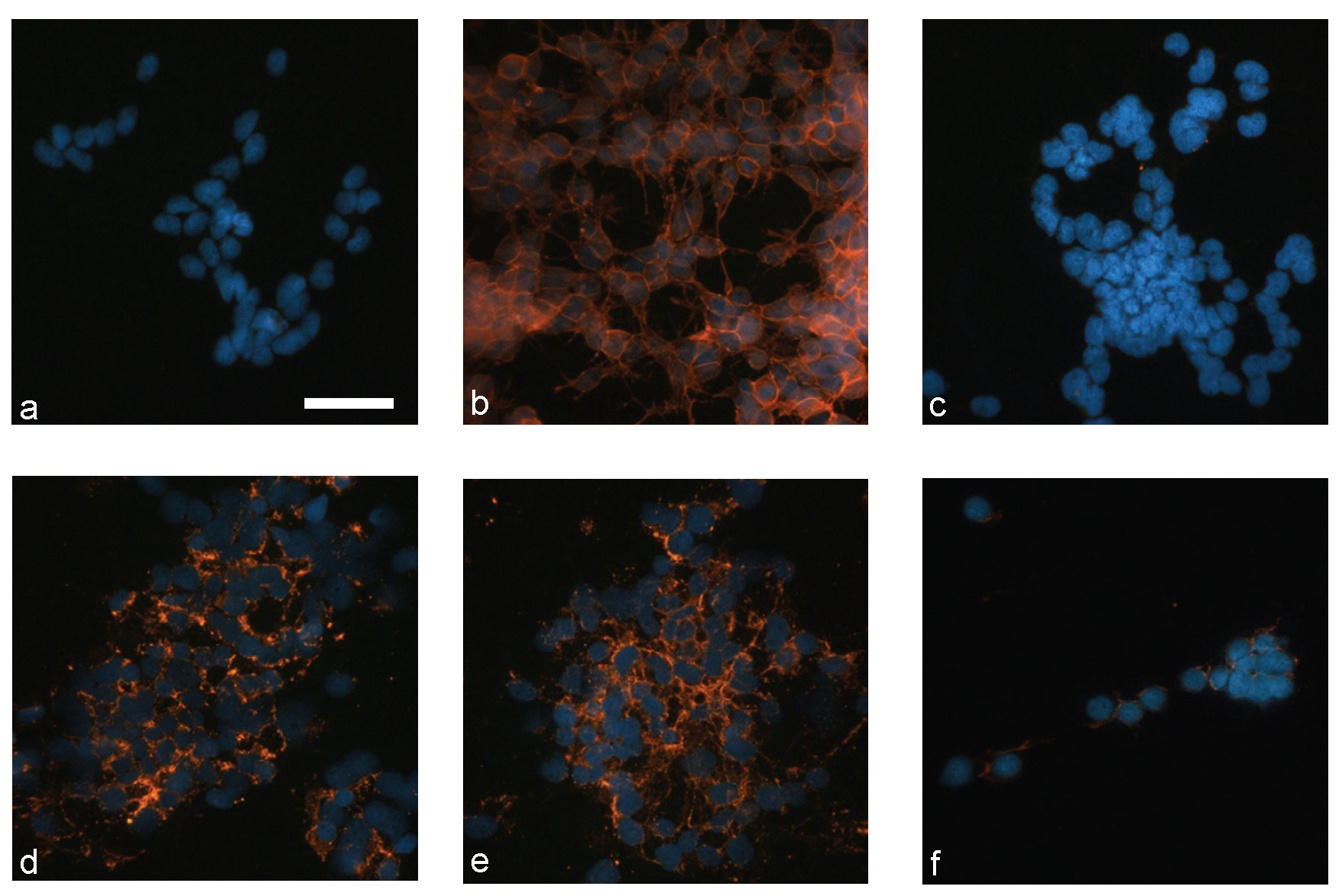

Supplement: Figure S2 — Effect of ST8SiaII inhibition on recovery of polySia expression following removal by EndoN in IMR-32 cells. IMR-32 cells immunolabelled with anti-polySia antibody (mAb 735) followed by incubation with TRITC-conjugated secondary antibody. (a) Negative control (absence of mAb 735); (b) Positive control (absence of endoN/CMP treatment) (c) Removal of polySia with EndoN (0.3 µg/mL); (d) PolySia recovery following 24 h incubation in absence of CMP; (e) PolySia recovery following 24 h incubation with CMP at 0.5 mM; (f) PolySia recovery following 24 h incubation with CMP at 5 mM. CMP clearly prevents the recovery of polySia on the cell surface following biological removal at 5 mM. (TIFF) [file pone.0073366.s002.tiff]

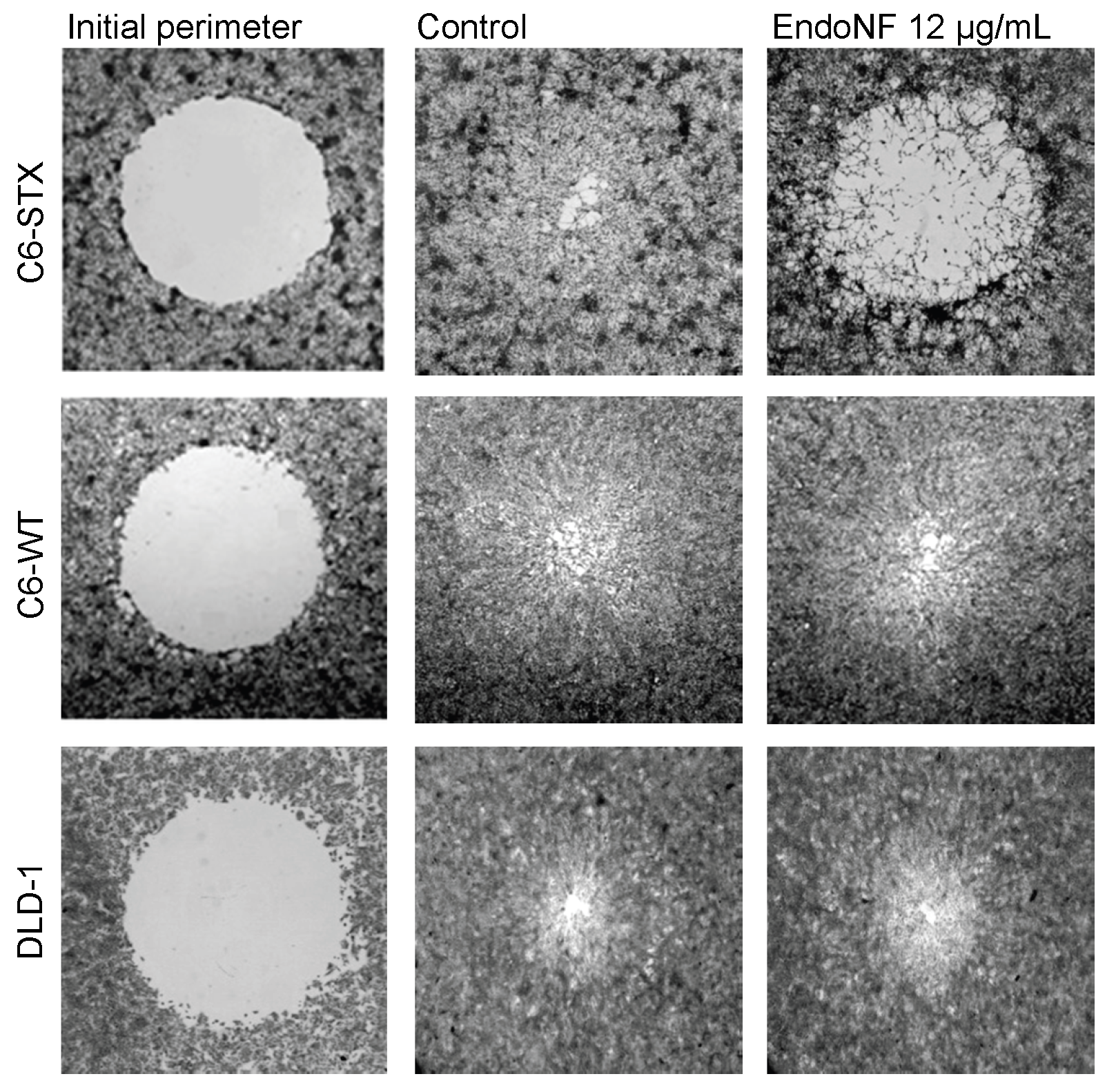

Supplement: Figure S3 — Effect of biological removal of polySia on tumor cell migration. Migration of C6-STX, C6-WT and DLD-1 cells was assessed. Confluent cell monolayers were incubated with fresh complete medium and repopulation of exclusion zones was assessed after 60 h for C6-STX cells, 72 h for C6-WT cells and 120 h for DLD-1 cells. EndoNF treatment of C6-STX cells led to a highly significant reduction in cell migration (17% of control, P<0.01), but no effect on C6-WT or DLD-1 cells. (TIFF) [file pone.0073366.s003.tiff]
